# The Syrian conflict: a case study of the challenges and acute need for medical humanitarian operations for women and children internally displaced persons

**DOI:** 10.1186/s12916-018-1041-7

**Published:** 2018-05-11

**Authors:** Rahma Aburas, Amina Najeeb, Laila Baageel, Tim K. Mackey

**Affiliations:** 1 Joint Masters Program in Health Policy and Law, University of California - California Western School of Law, San Diego, CA USA; 2 Brotherhood Medical Center for Women and Children, Atimah, Syria; 3 Department of Anesthesiology, University of California, San Diego School of Medicine, San Diego, CA USA; 4 Department of Medicine, Division of Global Public Health, University of California, San Diego School of Medicine, San Diego, CA USA; 5 Global Health Policy Institute, San Diego, CA USA

**Keywords:** Maternal child health, Syrian crisis, Humanitarian health aid, Internally displaced people

## Abstract

**Background:**

After 7 years of increasing conflict and violence, the Syrian civil war now constitutes the largest displacement crisis in the world, with more than 6 million people who have been internally displaced. Among this already-vulnerable population group, women and children face significant challenges associated with lack of adequate access to maternal and child health (MCH) services, threatening their lives along with their immediate and long-term health outcomes.

**Discussion:**

While several health and humanitarian aid organizations are working to improve the health and welfare of internally displaced Syrian women and children, there is an immediate need for local medical humanitarian interventions. Responding to this need, we describe the case study of the Brotherhood Medical Center (the “Center”), a local clinic that was initially established by private donors and later partnered with the Syrian Expatriate Medical Association to provide free MCH services to internally displaced Syrian women and children in the small Syrian border town of Atimah.

**Conclusions:**

The Center provides a unique contribution to the Syrian health and humanitarian crisis by focusing on providing MCH services to a targeted vulnerable population locally and through an established clinic. Hence, the Center complements efforts by larger international, regional, and local organizations that also are attempting to alleviate the suffering of Syrians victimized by this ongoing civil war. However, the long-term success of organizations like the Center relies on many factors including strategic partnership building, adjusting to logistical difficulties, and seeking sustainable sources of funding. Importantly, the lessons learned by the Center should serve as important principles in the design of future medical humanitarian interventions working directly in conflict zones, and should emphasize the need for better international cooperation and coordination to support local initiatives that serve victims where and when they need it the most.

## Background

The Syrian civil war is the epitome of a health and humanitarian crisis, as highlighted by recent chemical attacks in a Damascus suburb, impacting millions of people across Syria and leading to a mass migration of refugees seeking to escape this protracted and devastating conflict. After 7 long years of war, more than 6 million people are internally displaced within Syria — the largest displacement crisis in the world — and more than 5 million registered Syrian refugees have been relocated to neighboring countries [1, 2]. In total, this equates to an estimated six in ten Syrians who are now displaced from their homes [3].

Syrian internally displaced persons (IDPs) are individuals who continue to reside in a fractured Syrian state now comprising a patchwork of government- and opposition-held areas suffering from a breakdown in governance [4]. As the Syrian conflict continues, the number of IDPs and Syrian refugees continues to grow according to data from the United Nations High Commissioner for Refugees (UNHCR). This growth is continuing despite some borders surrounding Syria being closed and in part due to a rising birth rate in refugee camps [5, 6]. This creates acute challenges for neighboring/receiving countries in terms of ensuring adequate capacity to offer essential services such as food, water, housing, security, and specifically healthcare [4, 7, 8].

Though Syrian refugees and IDPs face similar difficulties in relation to healthcare access in a time of conflict and displacement, their specific challenges and health needs are distinctly different, as IDPs lack the same rights guaranteed under international law as refugees, and refugees have variations in access depending on their circumstances. Specifically, there are gaps in access to medical care and medicines for both the internally displaced and refugees, whether it be in Syria, in transit countries (including services for refugees living in camps versus those living near urban cities), or in eventual resettlement countries. In particular, treatment of chronic diseases and accessing of hospital care can be difficult, exacerbated by Syrian families depleting their savings, increased levels of debt, and a rise in those living in poverty (e.g., more than 50% of registered Syrian refugees in Jordan are burdened with debt) [9].

Despite ongoing actions of international humanitarian organizations and non-governmental organizations (NGOs) to alleviate these conditions, healthcare access and coverage for displaced Syrians and refugees is getting worse as the conflict continues [4, 10]. Although Syria operated a strong public health system and was experiencing improved population health outcomes pre-crisis, the ongoing conflict, violence, and political destabilization have led to its collapse [11–13]. Specifically, campaigns of violence against healthcare infrastructure and workers have led to the dismantling of the Syrian public health system, particularly in opposition-held areas, where access to even basic preventive services has been severely compromised [14–17].

Collectively, these dire conditions leave millions of already-vulnerable Syrians without access to essential healthcare services, a fundamental human right and one purportedly guaranteed to all Syrian citizens under its constitution [4]. Importantly, at the nexus of this health and humanitarian crisis are the most vulnerable: internally displaced Syrian women and children. Hence, this opinion piece first describes the unique challenges and needs faced by this vulnerable population and then describes the case study of the Brotherhood Medical Center (the “Center”), an organization established to provide free and accessible maternal and child health (MCH) services for Syrian IDPs, and how it represents lessons regarding the successes and ongoing challenges of a local medical humanitarian intervention.

### Syria: a health crisis of the vulnerable

Critically, women and children represent the majority of all Syrian IDPs and refugees, which directly impacts their need for essential MCH services [18]. Refugee and internally displaced women and children face similar health challenges in conflict situations, as they are often more vulnerable than other patient populations, with pregnant women and children at particularly high risk for poor health outcomes that can have significant short-term, long-term, and inter-generational health consequences [10]. Shared challenges include a lack of access to healthcare and MCH services, inadequate vaccination coverage, risk of malnutrition and starvation, increased burden of mental health issues due to exposure to trauma, and other forms of exploitation and violence such as early marriage, abuse, discrimination, and gender-based violence [4, 10, 19, 20]. Further, scarce medical resources are often focused on patients suffering from acute and severe injury and trauma, leading to de-prioritization of other critical services like MCH [4].

#### Risks for women

A 2016 United Nations Population Fund (UNFPA) report estimated that 360,000 Syrian IDPs are pregnant, yet many do not receive any antenatal or postnatal care [21, 22]. According to estimates by the UNFPA in 2015, without adequate international funding, 70,000 pregnant Syrian women faced the risk of giving birth in unsafe conditions if access to maternal health services was not improved [23]. For example, many women cannot access a safe place with an expert attendant for delivery and also may lack access to emergency obstetric care, family planning services, and birth control [4, 19, 24–28]. By contrast, during pre-conflict periods, Syrian women enjoyed access to standard antenatal care, and 96% of deliveries (whether at home or in hospitals) were assisted by a skilled birth attendant [13]. This coverage equated to improving population health outcomes, including data from the Syrian Ministry of Health reporting significant gains in life expectancy at birth (from 56 to 73.1 years), reductions in infant mortality (decrease from 132 per 1000 to 17.9 per 1000 live births), reductions in under-five mortality (from 164 to 21.4 per 1000 live births), and declines in maternal mortality (from 482 to 52 per 100,000 live births) between 1970 and 2009, respectively [13].

Post-conflict, Syrian women now have higher rates of poor pregnancy outcomes, including increased fetal mortality, low birth weights, premature labor, antenatal complications, and an increase in puerperal infections, as compared to pre-conflict periods [10, 13, 25, 26]. In general, standards for antenatal care are not being met [29]. Syrian IDPs therefore experience further childbirth complications such as hemorrhage and delivery/abortion complications and low utilization of family planning services [25, 28]. Another example of potential maternal risk is an alarming increase in births by caesarean section near armed conflict zones, as women elect for scheduled caesareans to avoid rushing to the hospital during unpredictable and often dangerous circumstances [10]. There is similar evidence from Syrian refugees in Lebanon, where rates of caesarean sections were 35% (of 6366 deliveries assessed) compared to approximately 15% as previously recorded in Syria and Lebanon [30].

#### Risks for children

Similar to the risks experienced by Syrian women, children are as vulnerable or potentially at higher risk during conflict and health and humanitarian crises. According to the UNHCR, there are 2.8 million children displaced in Syria out of a total of 6.5 million persons, and just under half (48%) of Syrian registered refugees are under 18 years old [1]. The United Nations Children’s Fund (UNICEF) further estimates that 6 million children still living in Syria are in need of humanitarian assistance and 420,000 children in besieged areas lack access to vital humanitarian aid [31].

For most Syrian internally displaced and refugee children, the consequences of facing lack of access to essential healthcare combined with the risk of malnutrition (including cases of severe malnutrition and death among children in besieged areas) represent a life-threatening challenge (though some studies have positively found low levels of global acute malnutrition in Syrian children refugee populations) [24, 32–34]. Additionally, UNICEF reports that pre-crisis 90% of Syrian children received routine vaccination, with this coverage now experiencing a dramatic decline to approximately 60% (though estimating vaccine coverage in Syrian IDP and refugee populations can be extremely difficult) [35]. A consequence of lack of adequate vaccine coverage is the rise of deadly preventable infectious diseases such as meningitis, measles, and even polio, which was eradicated in Syria in 1995, but has recently re-emerged [36–38]. Syrian refugee children are also showing symptoms of psychological trauma as a result of witnessing the war [4, 39].

## Discussion

### A local response: the Brotherhood Medical Center

In direct response to the acute needs faced by Syrian internally displaced women and children, we describe the establishment, services provided, and challenges faced by the Brotherhood Medical Center (recently renamed the Brotherhood Women and Children Specialist Center and hereinafter referred to as the “Center”), which opened its doors to patients in September 2014. The Center was the brainchild of a group of Syrian and Saudi physicians and donors who had the aim of building a medical facility to address the acute need for medical humanitarian assistance in the village of Atimah (Idlib Governorate, Syria), which is also home to a Syrian displacement camp.

Atimah (Idlib Governorate, Syria) is located on the Syrian side of the Syrian-Turkish border. Its population consisted of 250,000 people pre-conflict in an area of approximately 65 km^2^. Atimah and its adjacent areas are currently generally safe from the conflict, with both Atimah and the entire Idlib Governorate outside the control of the Syrian government and instead governed by the local government. However, continued displacement of Syrians seeking to flee the conflict has led to a continuous flow of Syrian families into the area, with the population of the town growing to approximately a million people.

In addition to the Center, there are multiple healthcare centers and field hospitals serving Atimah and surrounding areas that cover most medical specialties. These facilities are largely run by local and international health agencies including Medecins Sans Frontieres (MSF), Medical Relief for Syria, and Hand in Hand for Syria, among others. Despite the presence of these organizations, the health needs of IDPs exceeds the current availability of healthcare services, especially for MCH services, as the majority of the IDPs belong to this patient group. This acute need formed the basis for the project plan establishing the Center to serve the unique needs of Syrian internally displaced women and children.

#### Operation of the Center

The Center’s construction and furnishing took approximately 1 year after land was purchased for its facility, a fact underlining the urgency of building a permanent local physical infrastructure to meet healthcare needs during the midst of a conflict. Funds to support its construction originated from individual donors, Saudi business men, and a group of physicians. In this sense, the Center represents an externally funded humanitarian delivery model focused on serving a local population, with no official government, NGO, or international organization support for its initial establishment.

The facility’s primary focus is to serve Syrian women and children, but since its inception in 2014, the facility has grown to cater for an increasing number of IDPs and their diverse needs. When it opened, facility services were limited to offering only essential outpatient, gynecology, and obstetrics services, as well as operating a pediatric clinic. The staffing at the launch consisted of only three doctors, a midwife, a nurse, an administrative aid, and a housekeeper, but there now exist more than eight times this initial staff count. The staff operating the Center are all Syrians; some of them are from Atimah, but many also come from other places in Syria. The Center’s staff are qualified to a large extent, but still need further training and continuing medical education to most effectively provide services.

Though staffing and service provision has increased, the Center’s primary focus is on its unique contribution to internally displaced women and children. Expanded services includes a dental clinic 1 day per week, which is run by a dentist with the Health Affairs in Idlib Governorate, and has been delegated to cover the dental needs for the hospital patients*.* Importantly, the Center facility has no specific policy on patient eligibility, its desired patient catchment population/area, or patient admission, instead opting to accept all women and children patients, whether seeking routine or urgent medical care, and providing its services free of charge.

Instead of relying on patient-generated fees (which may be economically prohibitive given the high levels of debt experienced by IDPs) or government funding, the Center relies on its existing donor base for financing the salaries for its physicians and other staff as well as the facility operating costs. More than an estimated 300 patients per day have sought medical attention since its first day of operation, with the number of patients steadily increasing as the clinic has scaled up its services.

Initially the Center started with outpatient (OPD) cases only, and after its partnership with the Syrian Expatriate Medical Association (SEMA) (discussed below), inpatient care for both women and children began to be offered. Patients’ statistics for September 2017 reported 3993 OPD and emergency room visits and 315 inpatient admissions including 159 normal deliveries and 72 caesarean sections, 9 neonatal intensive care unit cases, and 75 admissions for other healthcare services. To better communicate the clinic’s efforts, the Center also operates a Facebook page highlighting its activities (in Arabic at https://www.facebook.com/مشفى-الإخاء-التخصصي-129966417490365/).

#### Challenges faced by the Center and its evolution

The first phase of the Center involved its launch and initial operation in 2014 supported by a small group of donors who self-funded the startup costs needed to operationalize the Center facility’s core clinical services. Less than 2 years later, the Center faced a growing demand for its services, a direct product of both its success in serving its targeted community and the protracted nature of the Syrian conflict. In other words, the Center facility has continuously needed to grow in the scope of its service delivery as increasing numbers of families, women, and children rely on the Center as their primary healthcare facility and access point.

Meeting this increasing need has been difficult given pragmatic operational challenges emblematic of conflict-driven zones, including difficulties in securing qualified and trained medical professionals for clinical services, financing problems involving securing funding due to the shutdown of banking and money transferring services to and from Syria, and macro political factors (such as the poor bilateral relationship between Syria and its neighboring countries) that adversely affect the clinic’s ability to procure medical and humanitarian support and supplies [40]. Specifically, the Center as a local healthcare facility originally had sufficient manpower and funding provided by its initial funders for its core operations and construction in its first year of operation. However, maintaining this support became difficult with the closure of the Syrian-Turkish border and obstacles in receiving remittances, necessitating the need for broader strategic partnership with a larger organization.

Collectively, these challenges required the management committee and leadership of the Center to shift its focus to securing long-term sustainability and scale-up of services by seeking out external forms of cooperation and support. Borne from this need was a strategic partnership with SEMA, designed to carry forward the next phase of the Center’s operation and development. SEMA, established in 2011, is a non-profit relief organization that works to provide and improve medical services in Syria without discrimination regarding gender, ethnic, or political affiliation — a mission that aligns with the institutional goals of the Center. Selection of SEMA as a partner was based on its activity in the region; SEMA plays an active role in healthcare provision in Idlib and surrounding areas. Some other organizations were also approached at the same time of this organization change, with SEMA being the most responsive.

Since the Center-SEMA partnership was consummated, the Center has received critical support in increasing its personnel capacity and access to medicines, supplies, and equipment, resulting in a gradual scale-up and improvement in its clinical services. This now includes expanded pediatric services and the dental clinic (as previously mentioned and important, as oral health is a concern for many Syrian parents and children). The Center also now offers caesarean deliveries [41]. However, the Center, similar to other medical humanitarian operations in the region, continues to face many financial and operational challenges, including shortage of medical supplies, lack of qualified medical personnel, and needs for staff development.

Challenges experienced by the Center and other humanitarian operations continue to be exacerbated by the ongoing threat of violence and instability emanating from the conflict that is often targeted at local organizations and international NGOs providing health aid. For example, MSF has previously been forced to suspend its operations in other parts of Syria, has evacuated its facilities after staff have been abducted and its facilities bombed, and it has also been subject to threats from terrorist groups like the Islamic State (IS) [42].

## Conclusions

The case study of the Center, which evolved from a rudimentary medical tent originally located directly in the Atimah displacement camp to the establishment of a local medical facility now serving thousands of Syrian IDPs, is just one example of several approaches aimed at alleviating the suffering of Syrian women and children who have been disproportionately victimized by this devastating health and humanitarian crisis. Importantly, the Center represents the maturation of a privately funded local operation designed to meet an acute community need for MCH services, but one that has necessitated continuous change and evolution as the Syrian conflict continues and conditions worsen. Despite certain successes, a number of challenges remain that limit the potential of the Center and other health humanitarian operations to fully serve the needs of Syrian IDPs, all of which should serve as cautionary principles for future local medical interventions in conflict situations.

A primary challenge is the myriad of logistical difficulties faced by local medical humanitarian organizations operating in conflict zones. Specifically, the Center continues to experience barriers in securing a reliable and consistent supply of medical equipment and materials needed to ensure continued operation of its clinical services, such as its blood bank, laboratory services, operating rooms, and intensive care units. Another challenge is securing the necessary funding to make improvements to physical infrastructure and hire additional staff to increase clinical capacity. Hence, though local initiatives like the Center may have initial success getting off the ground, scale-up and ensuring sustainability of services to meet the increasing needs of patients who remain in a perilous conflict-driven environment with few alternative means of access remain extremely challenging.

Despite these challenges, it is clear that different types of medical humanitarian interventions deployed in the midst of health crises have their own unique roles and contributions. This includes a broad scope of activities now focused on improving health outcomes for Syrian women and children that are being delivered by international aid agencies located outside of the country, international or local NGOs, multilateral health and development agencies, and forms of bilateral humanitarian assistance. The Center contributes to this health and humanitarian ecosystem by providing an intervention focused on the needs of Syrian women and children IDPs where they need it most, close to home.

However, the success of the Center and other initiatives working to end the suffering of Syrians ultimately relies on macro organizational and political issues outside Atimah’s border. This includes better coordination and cooperation of aid and humanitarian stakeholders and increased pressure from the international community to finally put an end to a civil war that has no winners — only victims — many of whom are unfortunately women and children.

## Abbreviations

the Center: the Brotherhood Women and Children Specialist Center
IDPs: Internally displaced persons
MCH: Maternal and child health
MSF: Medecins Sans Frontieres
NGOs: Non-governmental organizations
OPD: Outpatient department
SEMA: Syrian Expatriate Medical Association
UNFPA: United Nations Population Fund
UNHCR: the United Nations High Commissioner for Refugees
UNICEF: The United Nations Children’s Fund
